# Uniportal thoracoscopic left posterior basal segmentectomy using a posterior approach

**DOI:** 10.1111/1759-7714.14572

**Published:** 2022-07-11

**Authors:** Hitoshi Igai, Natsumi Matsuura, Mitsuhiro Kamiyoshihara

**Affiliations:** ^1^ Department of General Thoracic Surgery Japanese Red Cross Maebashi Hospital Maebashi Japan

**Keywords:** posterior approach, posterior basal segmentectomy, uniportal thoracoscopic approach

## Abstract

Posterior basal (S10) segmentectomy is one of the most challenging (and uncommon) types of pulmonary segmentectomy. Here, we present two key tips for facilitating a uniportal operation. The first is a full understanding of the relative locations of the pulmonary vessels and bronchi (as revealed by preoperative three‐dimensional computed tomography/broncho‐angiography), and the other is the use of “suction‐guided stapling” to dissect and divide the peripheral pulmonary vessels and bronchi. We describe the successful postoperative course of a patient who was so treated.

## Graphical abstract

Posterior basal (S10) segmentectomy is one of the most challenging (and uncommon) types of pulmonary segmentectomy. We here present two key tips, which are a full understanding of the relative locations of the pulmonary vessels and bronchi by preoperative three‐dimensional computed tomography/broncho‐angiography and the use of “suction‐guided stapling”, facilitating a uniportal operation.

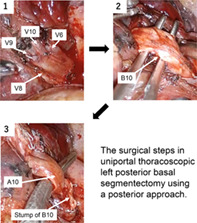

## INTRODUCTION

The results of JCOG0802/WJOG4607 by Saji et al. indicated that segmentectomy will become one of the standard procedures for lung cancer in the future.^1^ Posterior basal (S10) or lateral and posterior basal (S9 + 10) pulmonary segmentectomies are uncommon and one of the most challenging lung operations among complicated types of segmentectomies because they require exposure and recognition of the common basal pulmonary vein branches that lie deep in the lung parenchyma.^2^ In a previous report, we applied an intersegmental tunneling method during thoracoscopic S9 + 10 segmentectomy with a multiportal approach.^3^ However, this procedure is inappropriate for patients with dense fissures because exposure of the pulmonary artery (A10 or A9 + 10) at the fissure is essential despite the fact that division of the intersegmental plane between S6 and S9 + 10 after tunneling enables easy recognition of the common basal pulmonary vein branches. On the contrary, the multiportal posterior approach toward thoracoscopic S9 + 10 or S10 segmentectomy described in several previous reports is not sensitive to the fissural grade.^4^, ^5^, ^6^ Here, we describe how to perform uniportal thoracoscopic S10 segmentectomy via a posterior approach.

## CASE REPORT

**FIGURE 1:**
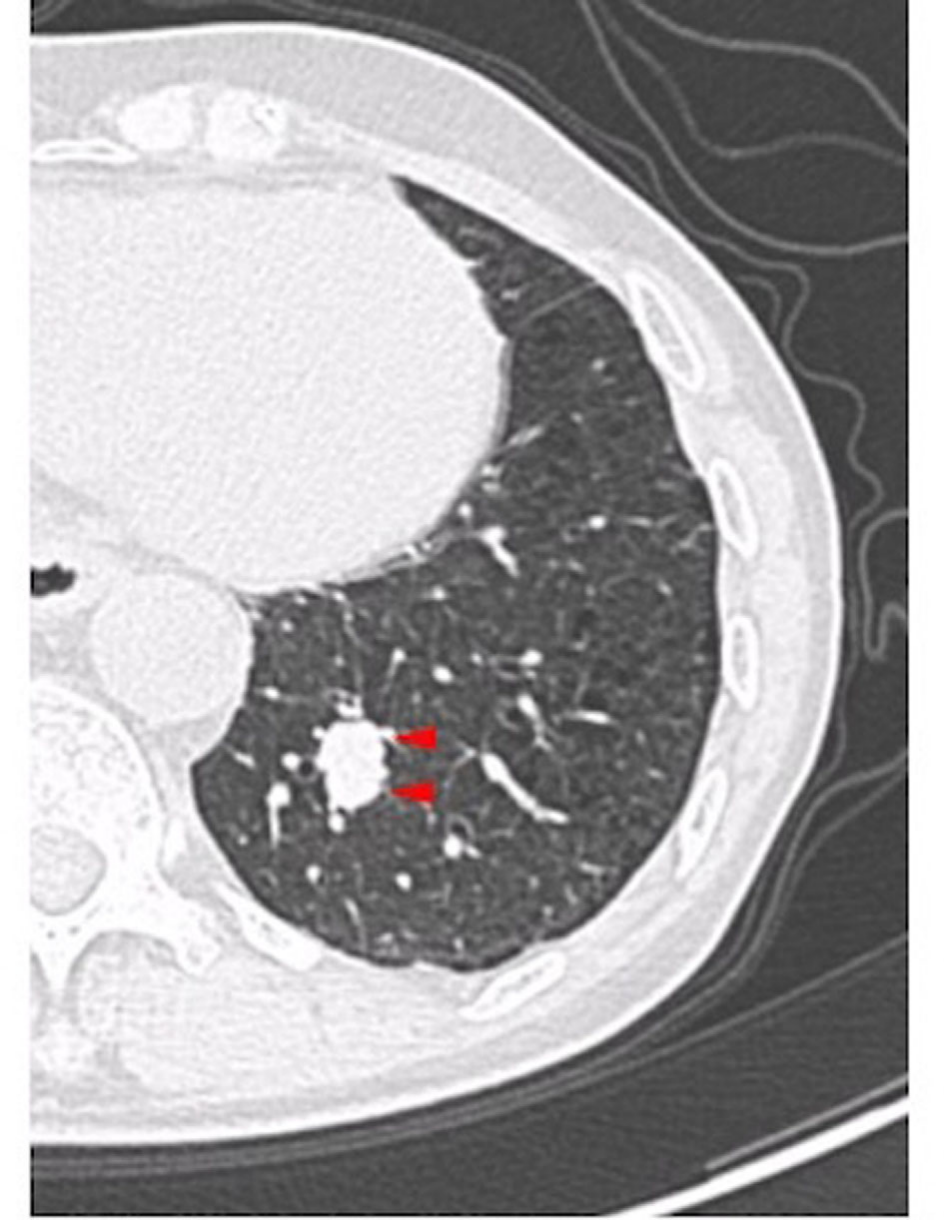
Preoperative computed tomography (CT) revealed a 20‐mm diameter solid nodular shadow (arrowheads) in the posterior basal (S10) segment, which was strongly suspected to be a pulmonary metastasis from colorectal cancer

**FIGURE 2:**
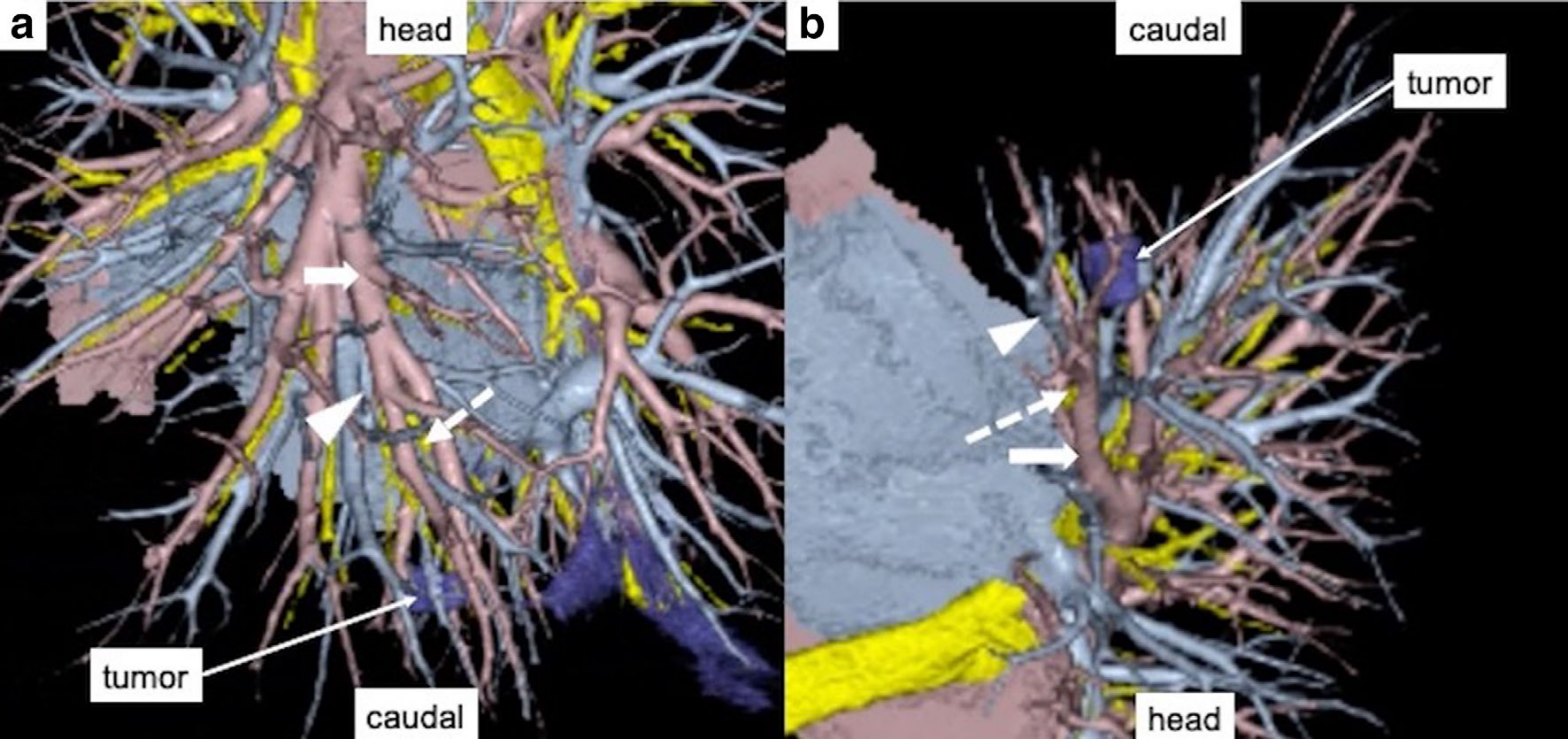
(a, b). Preoperative three‐dimensional CT broncho‐angiography revealed the relative locations of the pulmonary vessels and bronchi. Arrow: Posterior basal branch of the pulmonary artery; dotted arrow: Posterior basal bronchus; arrowhead: Posterior basal branch of the pulmonary vein

**FIGURE 3:**
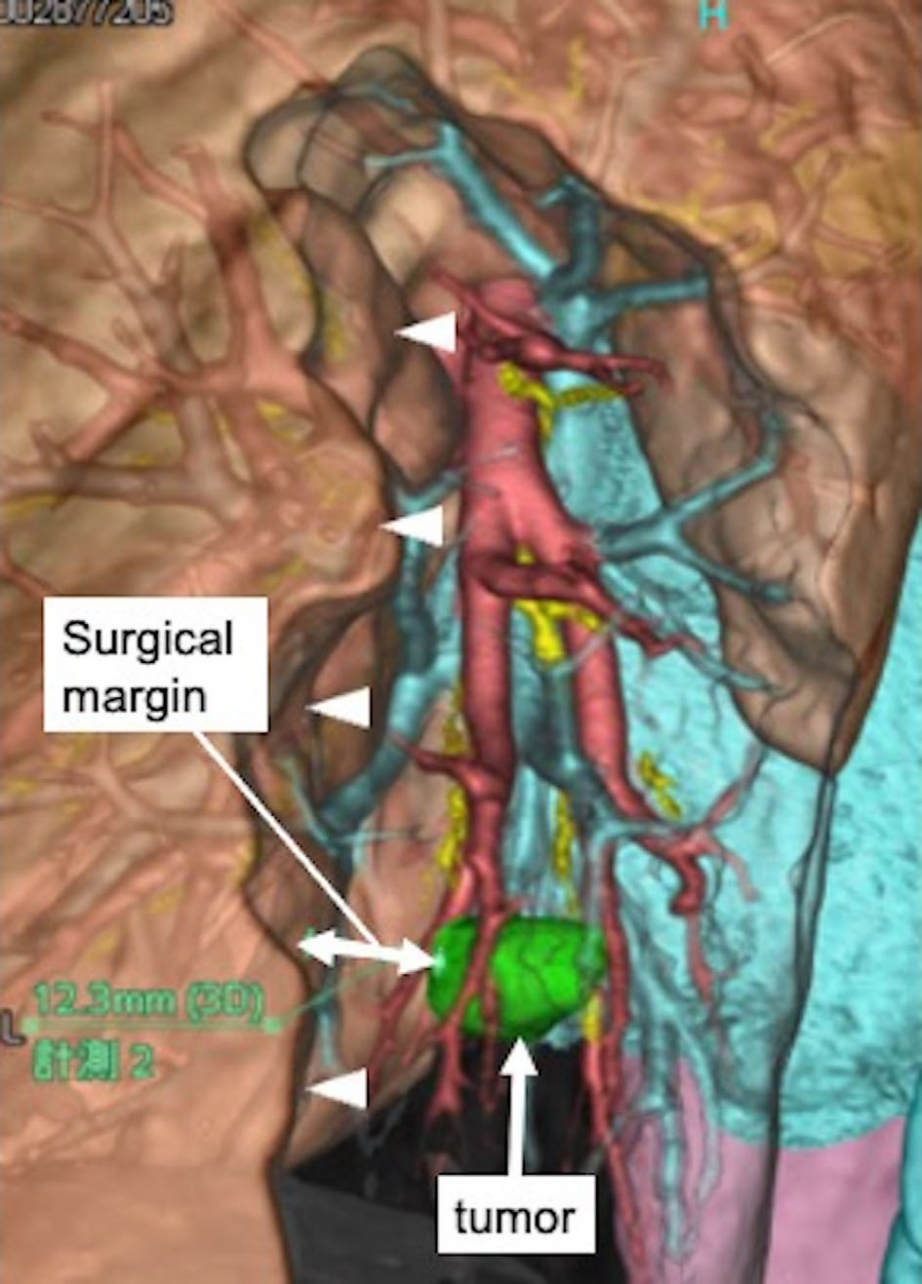
Preoperative simulation revealed that the target tumor would be included with a sufficient surgical margin by left posterior basal segmentectomy. The arrowheads indicate the intersegmental plane

A 74‐year‐old man with a history of colorectal cancer visited us because chest computed tomography (CT) revealed an approximately 20‐mm diameter solid nodular shadow in the posterior basal (S10) segment (Figure 1). Pulmonary metastasis was strongly suspected; there was no other distant metastasis. Therefore, uniportal thoracoscopic S10 segmentectomy (curative resection) was planned. Preoperative three‐dimensional CT/broncho‐angiography (3DCTBA) was performed to reveal the relative locations of the pulmonary vessels and bronchi (Figure 2). Moreover, sufficient surgical margin was ensured by left posterior basal segmentectomy by preoperative simulation (Figure 3).

The operation was performed with the patient under general anesthesia and one‐lung ventilation, in the lateral decubitus position. A 4‐cm access incision was created on the anterior axillary line of the fifth intercostal space. A 10‐mm 30° thoracoscope and other surgical instruments were inserted through that incision. The details are shown in a Video S1. We initially divided the posterior basal segmental vein (V10) (using a stapler) after incising the pulmonary ligament up to the inferior pulmonary vein. Then, the posterior basal segmental bronchus (B10) was fully exposed, and divided by a stapler, retracting the lower lobe toward the head. During exposure, the inflation‐deflation method was used to confirm that the bronchus was B10. Only S10 finally deflated, confirming that the bronchus was indeed B10. Next, the posterior basal segmental artery (A10) was exposed and divided using a stapler, with retraction of the B10 stump toward the head. The hilar tissue was dissected to create a space for the stapler that divided the intersegmental plane. After intravenous administration of indocyanine green, infrared thoracoscopic observation identified the plane between S10 and the other segments, which was divided using staplers. Finally, the specimen was removed via the thorax. The final pathology assessment revealed that the tumor was a pulmonary metastasis of the colorectal cancer. The postoperative chest drainage tube was removed on postoperative day 1 because there were no air leaks. The patient was discharged on postoperative day 5.

## DISCUSSION

We performed uniportal pulmonary S10 segmentectomy of the left lower lobe. There are two key tips. The first is that a full understanding of the relative locations of the pulmonary vessels and bronchi as revealed by preoperative 3DCTBA is necessary. Given that the intrathoracic structures are unidirectionally dissected, it is crucial to avoid confusion regarding the anatomy (especially by less‐experienced surgeons). Careful anatomical evaluation pre‐ and perioperatively is essential. In our department, we confirmed the location of pulmonary vessels and bronchi from several viewing by rotating the 3DCTBA data which was very helpful. The second tip is appropriate dissection and division of the peripheral pulmonary vessels and bronchi. When using a uniportal approach, it is sometimes difficult to dissect these tissues because it is not easy to grasp the surrounding tissues. Stapler insertion is also difficult (for the same reason). We highly recommend our “suction‐guided stapling” method for division of the pulmonary vein and bronchi.^7^ The space required for smooth stapling is created using only a long, curved suction device. This greatly aids uniportal surgery because the number of surgical instruments that can be inserted is limited. On the contrary, this surgical technique should not be adopted for division of the pulmonary artery because the wall of pulmonary artery is very fragile. Sufficient exposure and direct stapling without tape is considered better for division of the pulmonary artery.

Compared to our previously described “intersegmental tunneling” method, a posterior approach is not contraindicated by a dense fissure. We removed the chest drainage tube on postoperative day 1 because no dense fissure was dissected, and there was no air leakage. A uniportal posterior approach facilitates unidirectional dissection of intrathoracic structures; we earlier found that such dissection was easy even via a uniport.^8^ Therefore, if the surgical team carefully evaluates the anatomy and chooses an appropriate surgical technique, a uniportal approach is very useful when performing challenging segmentectomies, even on patients with dense fissures.

## CONFLICT OF INTEREST

None declared.

## Supporting information


**Video S1** Uniportal thoracoscopic left posterior basal segmentectomy using a posterior approach.
